# In Silico Evaluation of Hexamethylene Amiloride Derivatives as Potential Luminal Inhibitors of SARS-CoV-2 E Protein

**DOI:** 10.3390/ijms231810647

**Published:** 2022-09-13

**Authors:** Pouria H. Jalily, Horia Jalily Hasani, David Fedida

**Affiliations:** Department of Anesthesiology, Pharmacology and Therapeutics, Faculty of Medicine, University of British Columbia, Vancouver, BC V6T 1Z4, Canada

**Keywords:** SARS-CoV-2, envelop protein, hexamethylene amiloride derivatives, viroporins, steered molecular dynamics, molecular docking

## Abstract

The coronavirus E proteins are small membrane proteins found in the virus envelope of alpha and beta coronaviruses that have a high degree of overlap in their biochemical and functional properties despite minor sequence variations. The SARS-CoV-2 E is a 75-amino acid transmembrane protein capable of acting as an ion channel when assembled in a pentameric fashion. Various studies have found that hexamethylene amiloride (HMA) can inhibit the ion channel activity of the E protein in bilayers and also inhibit viral replication in cultured cells. Here, we use the available structural data in conjunction with homology modelling to build a comprehensive model of the E protein to assess potential binding sites and molecular interactions of HMA derivatives. Furthermore, we employed an iterative cycle of molecular modelling, extensive docking simulations, molecular dynamics and leveraging steered molecular dynamics to better understand the pore characteristics and quantify the affinity of the bound ligands. Results from this work highlight the potential of acylguanidines as blockers of the E protein and guide the development of subsequent small molecule inhibitors.

## 1. Introduction

Coronaviruses have been associated with deadly respiratory infections, and seven coronaviruses have been identified that infect and spread amongst humans. In the last two decades, the two epidemics caused by beta coronaviruses are Severe Acute Respiratory Syndrome coronavirus (SARS-CoV) and Middle East Respiratory Syndrome coronavirus (MERS-CoV). SARS-CoV-2 causes COVID-19 disease and is the most recently discovered strain of this coronavirus family which is responsible for the pandemic that started in late 2019 in the city of Wuhan, China [1,2,3,4,5,6,7,8]. The SARS-CoV-2 genome encodes a viral replicase encompassing structural proteins that include Spike (S), Envelope (E), Membrane (M) and Nucleocapsid (N), and a group of accessory proteins specific to SARS-CoV-2 *viz*. 3a, 3b, 6, 7a, 7b, 8b, 9b and 10 [9]. The S and M proteins form spikes on the surface that project 17–20 nm [10].

Another important component of the viral envelope is the E protein which is present on the surface of the virion in smaller numbers [5,11]. This E protein is the least studied and understood of the four structural proteins encoded by SARS-CoV-1 and SARS-CoV-2, but is thought to encode a 75-amino acid transmembrane-spanning protein that acts as a viral ion channel or viroporin, and plays a crucial role in viral assembly, budding, virion release, and viral pathogenesis, to the extent that E protein knockout coronaviruses are considered as putative vaccine candidates [12,13,14,15,16]. Recently, through a set of in vitro and in vivo studies, Xia et al. reported that the SARS-CoV-2 E protein alone causes acute respiratory distress syndrome (ARDS)-like damage, suggesting inhibition of the E protein as a promising antiviral strategy [17].

The E protein of SARS-CoV-2 has a short hydrophobic N-terminus comprised of 7-12 residues which is connected to a 25-amino acid Trans-Membrane Domain (TMD) and terminates in a comparatively long hydrophilic C-terminus [10,11,18,19,20]. The TMD has a prevalence (~35%) of valine and leucine residues which are responsible for the high grand average hydropathicity index of this protein. The C-terminal tail contains cysteines and prolines that act as palmitoylation targets and Golgi-complex targeting signals, respectively, and mutation of the prolines prevents the localization of the E protein in Golgi complexes and instead, the mutant E protein populates the plasma membrane [11,21].

Transient expression of N- or C-terminally flagged E protein of SARS-CoV-1 showed both termini present in the cytoplasm, supporting a hairpin topology [22]. However, in infected and transiently transfected cells, the SARS-CoV-1 E protein adopts a cytoplasmic C-terminal and endoplasmic N-terminal orientation [23]. More recently, Duart et al. demonstrated that the SARS-CoV-2 E protein is a single TMD-spanning membrane protein with cytoplasmic C-terminal and endoplasmic N-terminal orientation in mammalian cells which supports the ion channel activity described by Verdiá-Báguena and coworkers and with the structural model of the E protein reported in micelles [24,25].

### 1.1. SARS-CoV-2 E Protein as a Viroporin

Viroporins or viral ion channels have been known since 1992 when the M2 ion channel of influenza A was discovered [26]. Viroporins comprise at least one TMD that spans the lipid bilayer in the form of a pore-forming homotetramer such as M2, or homopentamer such as the E protein [13,27,28] which can alter membrane potential [23,29,30]. Several studies have confirmed the homopentameric arrangement of the coronavirus E proteins, including a sedimentation equilibrium study in dodecyl phosphocholine micelles [31] and isotopically labelled peptides reconstituted in model lipid bilayers and detergent micelles found to also be consistent with a homopentameric model [27,32].

The E proteins of SARS-CoV-1 [13,14,28,31,33,34,35], HCoV-229E [34] and avian infectious bronchitis virus (IBV) [36] exhibit electrophysiological activity and act as cation-selective ion channels that can be inhibited by HMA [18,20,28,34]. Changes in the ion concentration in the host cells are required for the production and maturation of viral progeny, but the role of ion channel activity in pathogenesis is not well understood.

In previous studies, the ion channel activity of the SARS-CoV-1 E protein was mapped within the TMD using synthetic peptides [13,14,33], and it was further suggested that the charge and nature of the lipid microenvironment contributed to the chemical environment of the lumen of the pore and affected ion conductance [37]. Cationic over anionic conductance was preferred when the chemically synthesized SARS-CoV-1 E protein was reconstituted in lipids that resembled the charge and composition of the ER-Golgi intermediate compartment (ERGIC) membranes, but no specific selectivity was exhibited for a particular cation. Additionally, Asn15Ala and Val25Phe were identified as loss-of-function mutations and were experimentally confirmed to suppress E protein channel activity [14,33].

Patch clamp recordings in HEK293 cells and two electrode-voltage clamp experiments in oocytes have shown SARS-CoV-2 ion channel activity at pH 6.0 and 7.4, and the E protein to be permeable to monovalent cations such as Na^+^, Cs^+^ and K^+^ [16,38]. Similarly, single channel electrophysiological recordings using planar lipid bilayers made by Xia et al. confirmed that: (i) SARS-CoV-2 E protein was permeable to Na^+^ and K^+^ but not to Cl^−^; and (ii) permeability of Na^+^ was equal to K^+^ but higher than divalent Ca^2+^ and Mg^2+^ [17].

### 1.2. HMA and HMA Derivatives as SARS-CoV2 E Protein Blockers

Historically, HMA has been shown to inhibit SARS-CoV-1 E protein ion channel activity [27,28,39,40,41] and SARS-CoV-2 E protein-mediated currents using patch clamp [20]. NMR studies show that HMA binds to hydrophilic residues in the lumen of the TMD close to the N terminus of the E protein [19]. The presence of HMA and amantadine has created chemical shift perturbations in 2D correlation spectra (^15^N-^13^C_α_ and ^13^C-^13^C), and specifically, residues Thr9, Gly10, Thr11, Ile13 and Ser16 were involved in HMA binding while mutation of Asn15 to alanine prevented binding of blockers at the N-terminal endoplasmic end of the channel. Binding affinities of EIPA and HMA were similar and their antiviral potencies in cell-based assays were greater than amiloride, suggesting that the presence of bulky aliphatic or aromatic substituents at the 5′-position of the pyrazine ring augment drug binding and antiviral activity [18].

In the present study, we have screened a panel of HMA derivatives with various chemical substituents and previously reported viroporin inhibitors against the E protein and further quantitatively calculated their activity relative to classical inhibitors such as HMA and amantadine through in silico analysis. Through a set of computational assessments in this study, including docking studies, MD simulations, binding free energy calculations and SMD simulations, we have confirmed that HMA derivatives 37, 33, 26 and 27 are able to bind to an endoplasmic site in the pore of the E protein. Physical occlusion of the pore was observed that can potentially interfere with cationic ion passage. Furthermore, we were also able to elucidate structural information and details about the pore characteristics secondary to the binding of the drug molecules.

## 2. Results and Discussion

### 2.1. Construction of the SARS-CoV-2 E Protein

An ssNMR structure of SARS-CoV-2 that spanned from residue 8–38 has been resolved (with reference to UNIPROT ID #P0DTC4; PDB ID: 7K3G) [19]. This structure lacks the C-terminal ending of the protein that is reported to adapt a helical secondary structure in the NMR spectral data, as found by Park et al. [18]. We addressed this structural insufficiency, by homology modelling the C-terminal helical arm using the structure of the SARS-CoV-1 E protein (PDB ID: 5X29) as the template (sequence similarity of 94.74%) and fusing it to the ssNMR structure of SARS-CoV-2 spanning the transmembrane region (PDB ID: 7K3G). Thereby generating a more comprehensive model with detailed structural data to be used in this study.

The Ramachandran plot of the chosen model shows 77.8% of residues in the favoured region and 22.2% of the residues in the allowed regions with very few outliers. The best local quality belongs to the transmembrane helix (residues 10–38) which acts as the main functional component of the protein. The middle helical segment (residues 39–48) and the C-terminal domain (residues 54–65) possess a slightly lower local quality, but still in the acceptable range. The assembly of the two protein segments, i.e., the transmembrane helix and C-terminal arm, was performed using the Build Structure module in Chimera suite wherein a peptide bond was built between Arg38 and Leu39 and minimized for 500 steps to ensure the removal of any structural clashes and/or strains.

### 2.2. Small Molecule Docking Simulations

The approximation of the drug binding site and the selection of the receptor grid for the purpose of this docking study were based on previous experimental ssNMR data [18,19]. The 2D correlation spectra (^15^N-^13^C_α_ and ^13^C-^13^C) from these NMR studies showed chemical shift perturbations suggesting that the HMA binding site is situated near the N-terminal residues with emphasis on residues Glu8, Thr9, Thr11 and Asn15 that were affected by ligand binding. Hence, we confined our search space during the multiple docking stages to a box of 20 Å^3^ in dimension. Initially, the SP docking was performed to determine the preliminary placement of the ligands at the chosen docking site.

The top poses to move forward were chosen based on their Glide docking scoring function in addition to visual inspection of poses and pore blockade. Accordingly, the resulting top 162 poses from the SP docking were subjected to XP docking with individually scored descriptors. Subsequently, the filtered poses of the more rigorous XP docking results led to 60 hits. Next, Schrodinger’s Induced Fit docking (IFD) module was used to refine and subsequently identify the best-scored ligands from XP docking with extended sampling. This led to ligand structures and conformations that were induced fit to the docking site.

These results were also filtered based on relevance to the available binding data from the literature, i.e., the presence of interactions with residues spanning the N-terminus of the pore lumen. The IFD docking scores of the final top 12 ligand poses along with the control molecules (amantadine, HMA and EIPA) are listed in Table 1. The inclusion of the control molecules enabled us to study the ligands relative to other reported E protein channel inhibitors. The docking scores of these final 12 poses ranged from −10.02 to −3.66 kcal/mol. Compound 26 showed the best score and amantadine showed the worst score. HMA derivatives ranked in the median range of −8 to −4 kcal/mol while only 26 and 27 had better docking scores. HMA and its closest derivative EIPA were scored poorly with values of −5.32 and −4.03 kcal/mol, respectively. 

Scoring functions are an effective tool to determine the overall binding mode and to predict the relative binding affinity of ligands for a given protein target, while methods that capture longer dynamicity of such protein–ligand interactions can offer a more in-depth look and provide more accurate predictions. The following section describes results obtained through a variety of molecular dynamic simulations as well as MM/GBSA binding free energy calculations and pore analyses.

### 2.3. Molecular Dynamics Simulation Studies

**Figure 1:**
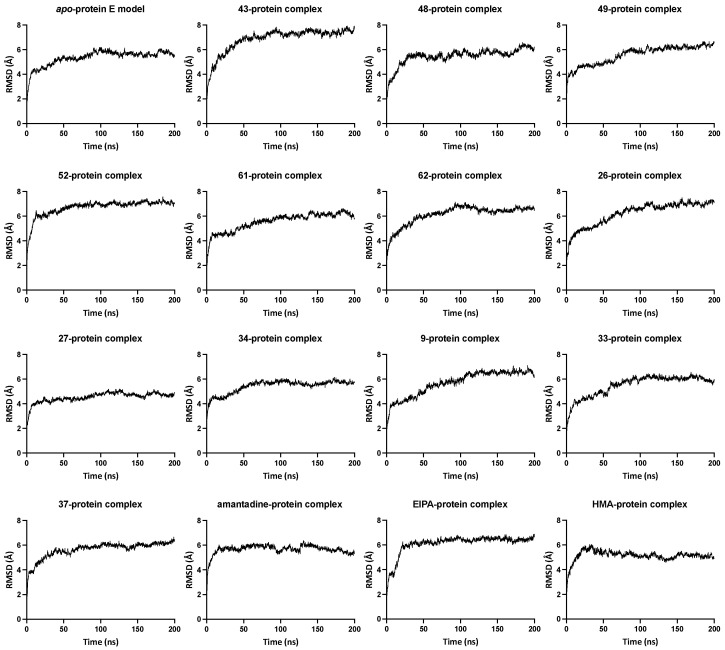
RMSD graphs of the protein in *apo*-state and ligand-bound state. The relative mean square deviation in Å for each protein complex system was measured with reference to the starting configuration plotted versus time. The number in each graph title refers to the compound number from Table 1.

Valuable information can be derived by comparing the structural drifts of the protein in the *apo*-state as well as in drug-bound complexes through RMSD analysis of the C-alpha atoms of the protein backbone throughout the production run of the MD simulation. In the majority of the simulations reported here, the RMSD values initially increased while the system was equilibrating and converging, at about the second half of the 200 ns long simulations. Data in Figure 1 illustrate the RMSD graphs of the protein in the *apo*-state as well as the ligand-bound state. The protein system demonstrates convergence starting at about 70 ns into the production run. Visual analysis of the MD trajectory showed structural stability and the protein retaining its pentameric integrity.

The RMSD of the ligand-bound protein shows a similar trend, i.e., convergence after about 50–75 ns. A few of the systems display a surge in the RMSD values at a certain stage of the MD simulation, indicating a form of temporary turbulence either in the pentameric state or instability caused by the ligand at the binding site, i.e., dislodging of the drug molecule or a rotation in the mode of binding. An example of these occurrences is displayed by the compound 37-protein complex in which one of the protein chains hinges near Asn15, caused by the ligand interactions (hydrogen bonding) with Val14, Val17 and Leu19 from two different helix chains entering into a sandwich conformation. However, the protein pushes the ligand back into the centre of the lumen, flipping between the two binding poses and causing instability in the system. The ligand occupancy is significantly higher in the central lumen than in the sandwich mode, which could be explained by the fact that the protein structure is quite robust and retains its pentameric arrangement and integrity even in the presence of an external entity.

**Figure 2:**
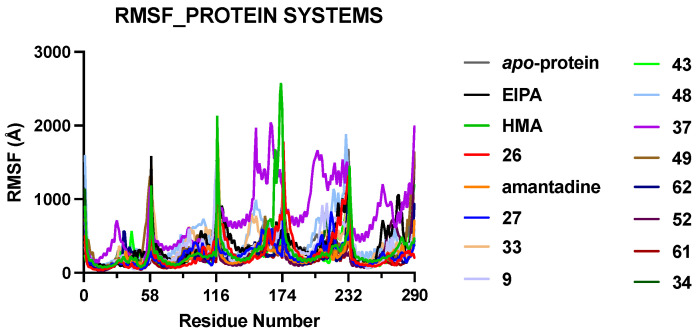
RMSF graphs of the five chains in the model protein systems. The protein systems are presented sequentially and labelled by the respective bound ligands. The highest degree of fluctuation was observed at the C-terminal end of the TM helix of each monomer within the protein–ligand complex.

**Figure 3:**
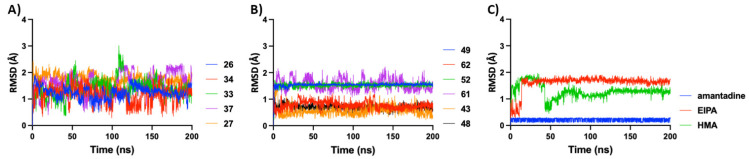
RMSD graphs of different ligands during MD simulations, overlayed and grouped based on their parent chemical structure. (**A**) Molecules with lower molecular weight, and more linear in shape exhibited a higher degree of deviation. (**B**) Larger molecules with higher degrees of derivatization were more stable when bound to E protein during 200 ns MD simulation. (**C**) Varying degree of deviation was observed for our control molecules.

Analyses of fluctuations as measured by RMSF calculations (Figure 2) reveal the degree of flexibility experienced by different parts of the protein throughout the MD simulations. We observed that the protein C-terminal end of the helical arm exhibited more fluctuations compared to the rest of the systems, leading to spikes in the RMSF values. The individual graphs of the RMSF value for different systems are shown in Figure S1. To gain a better understanding of atomic-level interactions between the E protein and HMA derivatives (listed in Table S1), we performed individualized structural analyses. These derivatives share an identical scaffold, and their difference lies in the substituents at the 5′ position of the pyrazine ring. Ligand RMSD values were plotted for all the top hits (Table 1) in our study presented in Figure 3 and used to check for fluctuations that could indicate instability in our MD simulations, or alternatively could provide insight into the characteristics of the binding. 

For the purpose of comparing the different ligands in this study, molecules were grouped into three main categories: the control group (amantadine, HMA and EIPA), compounds 9, 11, 22, 26, 27, 33–38 and 39–62. As shown in Figure 3, compounds’ (9, 11, 22, 26, 27 and 33–38) RMSD values fluctuate between 0.5–2.5 Å throughout MD simulations. This group possesses relatively higher flexibility through rotation around the C-C bond linking the two substituted aromatic rings. Compounds 39–62, however, exhibit a different pattern such that some of the ligands had an average RMSD value of less than 1.0 Å, whereas others showed more fluctuation (albeit with a similar variance) throughout the 200 ns MD simulation. This can be explained by the fact that the ligands in the latter group were bulkier in size and lacked rotatable intramolecular bonds which would in turn limit molecular flexibility. A similar trend is observed in the control group where our smallest ligand i.e., amantadine, which lacks flexible bonds, exhibits a more stable state and the deviations are minimal, but HMA and EIPA owing to their bigger size and presence of multiple rotatable bonds exhibited a higher degree of deviation. 

The shifts observed in RMSD graphs of HMA and EIPA suggest two possible binding modes. HMA initially binds at the center of the lumen symmetrically interacting with residues from all five protein chains. However, about 40 ns into the MD simulation, the ligand finds itself in a more stable binding pocket cavity formed by three adjacent chains and retains this pose for the remainder of the trajectory. This flip in the binding pose is responsible for the sudden shift in RMSD values from 2.0 Å down to 0.5 Å and thereafter converging at 1.3 Å. Interestingly, a similar trend was observed for EIPA at a much earlier stage of the MD simulation. The individual RMSD graphs of all the ligands are presented in Figure S2. 

To differentiate and rank the ligands based on their binding affinities, the MM/GBSA technique in AMBER was utilized [43,44]. The binding free energy values of the molecules in the study are listed in Table 1 along with their corresponding docking scores. Positive controls such as HMA and EIPA showed mid-range values of −25.46 and −27.51 kcal/mol, respectively, whereas 26 and 27 along with 33 and 61 possess energies in the range of −32 up to −40 kcal/mol, making these the strongest binding ligands in this study. The weaker-bound ligands are 9, 34 and amantadine, whose binding energies lie between the −8 to −11 kcal/mol range. 

### 2.4. In-Depth Analysis of the Intermolecular Interactions between the Ligands and the E Protein

**Figure 4:**
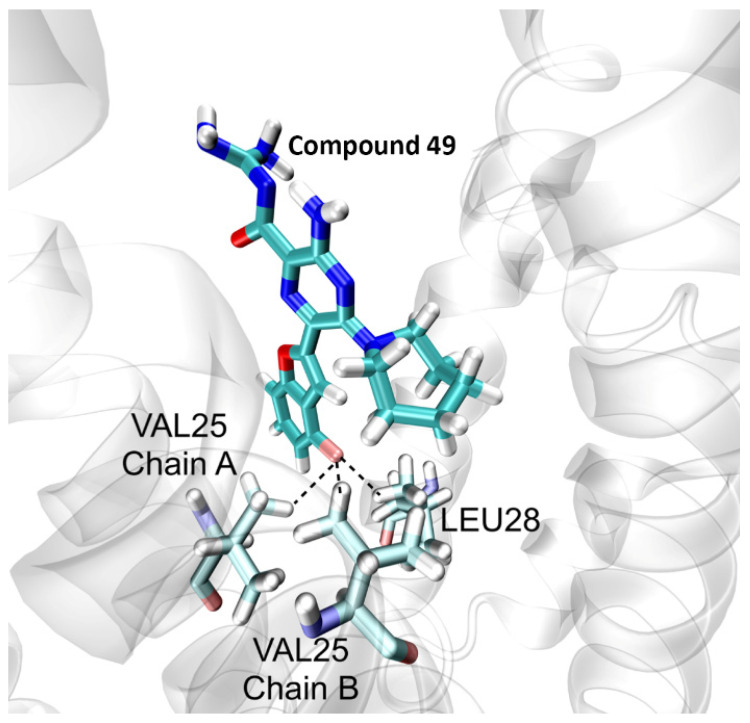
Interaction of the fluoro-substituted benzofuran of compound 49 with the E protein. The figure shows the stable conformation of the ligand forming tridentate hydrogen bond interactions with Val25 and Leu28.

Compound 49 possesses a fluoro-benzofuran ring which forms tridentate hydrogen bonds with Leu28 and two Val25 residues from two different chains, as shown in Figure 4, which stabilizes the ligand at the centre of the lumen and prevents structural drifts in its placement as well as the protein. This may explain why 49 possesses a more stable RMSD as compared to a ligand such as 48 which has an indole substituent group at the 5′ position. The latter does not form significant hydrogen bond interactions with the pore lining residues and therefore is not as stable and robustly bound during the MD trajectory.

**Figure 5:**
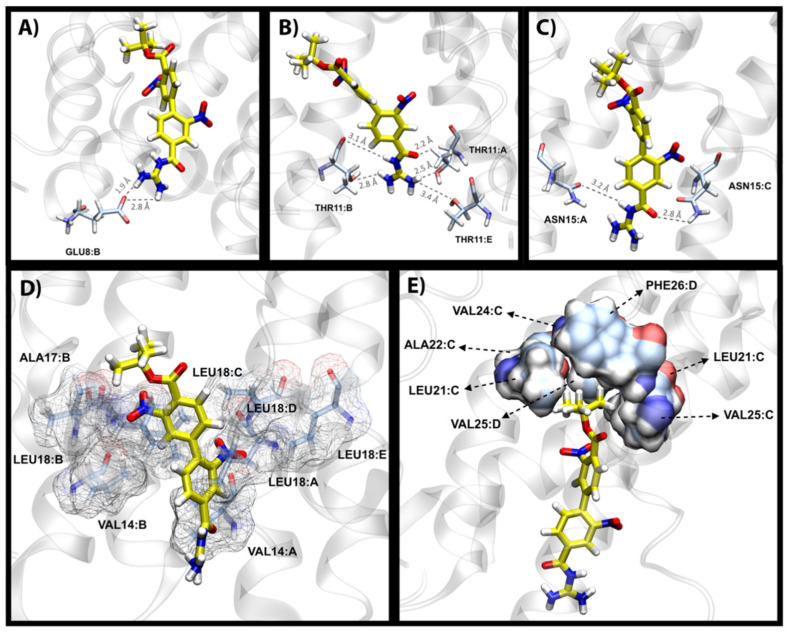
Overview of the different types of protein–ligand interactions of compound 27. Hydrogen bond formations between the iso-guanidine moiety of 27 and Glu8 (**A**), Thr11 (**B**) and Asn15 (**C**) are shown from top left to right. For clarity of presentation, only one or two residues from the respective chains are shown. However, it should be noted that interactions of such nature occur through a toggling process where the ligand interchangeably interacts with all chains. (**D**) The belt formation of hydrophobic residues: Val14, Ala17 and Leu18 around the aromatic rings of 27. (**E**) Hydrophobic umbrella-shaped pocket interactions of 27’s BOC moiety interacting with Leu21, Ala22, Val24, Val25 and Phe26.

Next, we investigated the binding mode of a top hit molecule, i.e., 27. Upon analyzing the networks of hydrogen bonds formed during MD simulations, several groups of residues were involved in forming a significant exchange of hydrogens with our ligand. Such analyses showed that the iso-guanidine moiety of 27 formed significant hydrogen bonds with Glu8 and Thr11 having 39.5% and 34.3% occupancy, respectively, during 200 ns MD simulations. The hydrogen bond with Asn15 had a higher occupancy of 71.6%, which indicates a prominent non-hydrophobic interaction with 27 that helps stabilize the core of the molecule. The examples of these three groups of hydrogen bonds are shown in Figure 5A–C, which agree with the solution NMR results from the study by Park et al. in regards to the significance of Glu8, Thr11 and Asn15 in ligand binding [18].

The second group of residues that are involved in ligand binding include the hydrophobic residues Val14, Ala17 and Leu18 shown in Figure 5D. These residues from different protein chains aggregate into a so-called belt formation around the middle section of the ligand. The nature of these interactions mainly involves the stabilization of the two aromatic rings of 27 through NH-π interactions and van der Waals forces. Interestingly, Leu18 of all five homomers of the protein consistently face the lining of the pocket at all times as opposed to other interacting residues where only two or three chains were involved at any given time, which supports a significant role for Leu18 in the binding of 27.

The BOC moiety of 27 is mainly involved in hydrophobic interactions with a well-defined umbrella-shaped pocket as shown in Figure 5E. Compound 27 possesses an angled orientation and rests the carbon-rich hydrophobic end of the molecule (BOC moiety) in a pocket which comprises Leu21, Ala22, Val24, Val25 and Phe26. Our channel pore analysis (see Section 2.6) reveals that residues Asn15, Leu18, Leu21 and Leu28 are responsible for the main constrictions in the lumen of the *apo*-E protein, and the presence of 27 at this position leads to further distortion of the ion passage pathway, which may result in the blockade by 27.

### 2.5. SMD Studies Using Na^+^

To gain a better understanding of pore characteristics and to quantify the affinity of the bound ligands, SMD studies were performed for three categories of molecules *viz.* control group (amantadine, HMA and EIPA), which exhibit different free binding energies, weak blockers (9 and 34) and strong blockers (26 and 27) in addition to the *apo*-protein. As described in the Materials and Methods, SMD simulation involves pulling an external entity, in this case, a Na^+^ from the C-terminal end of the TM helix to the periphery of the N-terminus along the *Z*-axis. The Na^+^ interacts with various pore-lining residues as well as the bound ligand in its path. The changing forces exerted on the SMD ion (Na^+^ in our case) are captured and plotted as a force profile which offers insight into the pore characteristics, the effect of ligand ion passage and different chemical environments along the protein pore lumen. For our SMD study, we chose representatives from each group of ligands based on their binding free energy. As such, 26 and 27 were chosen as the strong ligands and 9 and 34 as our weak ligands. To allow for a comparison, we also included the three control ligands in our SMD study, which happen to exhibit different free binding energy amongst themselves. Additionally, we also pulled a Na^+^ through the *apo*-protein system to study pore characteristics in the unbound state. Detailed analyses from our SMD studies will be discussed in the following section.

**Figure 6:**
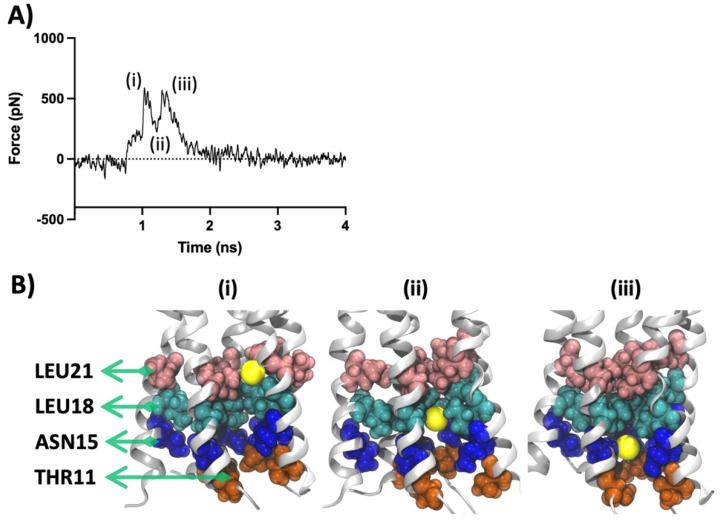
SMD results for unbound *apo*-protein. (**A**) SMD force profile depicting the different peaks labelled (i–iii). The first peak (i) represents the constriction formed by Leu18 and Leu21 followed by a trough formation (ii). The next twin-peak formation (iii) represents the obstacle formed by the cluster of Asn15 and Thr11 residues. (**B**) Three snapshots representing the significant events in the force profile are also respectively labelled (i–iii). The lateral view is along the *Z*-axis of the protein. Protein is shown in silver cartoon representation and the residues are shown with VDW representation. The sodium ion is shown in yellow as VDW representation.

Each peak in the SMD force profiles represents an energetic obstruction along the movement path of the SMD ion. By quantifying the forces experienced by the SMD ion, a better understanding of the nature, as well as the strength of interactions formed between the ligand–protein, protein–protein, and ion–ligand/protein can be achieved. The force profile of the SMD simulation of the *apo*-protein when the Na^+^ is pulled through the unbound protein model is shown in Figure 6.

Two peaks represent the main barriers on the path of the SMD ion movement. The first peak (i) is the result of the obstruction created by the collective opposing forces (~530 pN) imposed by clusters of Leu21 and Leu18 residues. These two sets of residues are among the pore-constricting components found in our HOLE pore analysis (see Section 2.6). As the Na^+^ overcomes this initial constriction, the release of Na^+^ creates a push and leads to a trough (ii) in the force profile. Furthermore, as the Na^+^ continues to travel down the pore, it comes across the impeding plane of Asn15 and Thr11 which leads to a distinct twin-peak formation. The three events described here are depicted in Figure 6B as three snapshots showing the position of the Na^+^ respective to the involved residues. This observation correlates with previous findings in the literature regarding the significance of Thr11, Asn15, Leu18 and Leu21 in ion conduction and ion selectivity as well as ligand binding [19]. Our ligand interaction analysis, discussed in the previous section, also highlights the role of these residues in forming a stable environment required for the binding of the channel blockers. 

**Figure 7:**
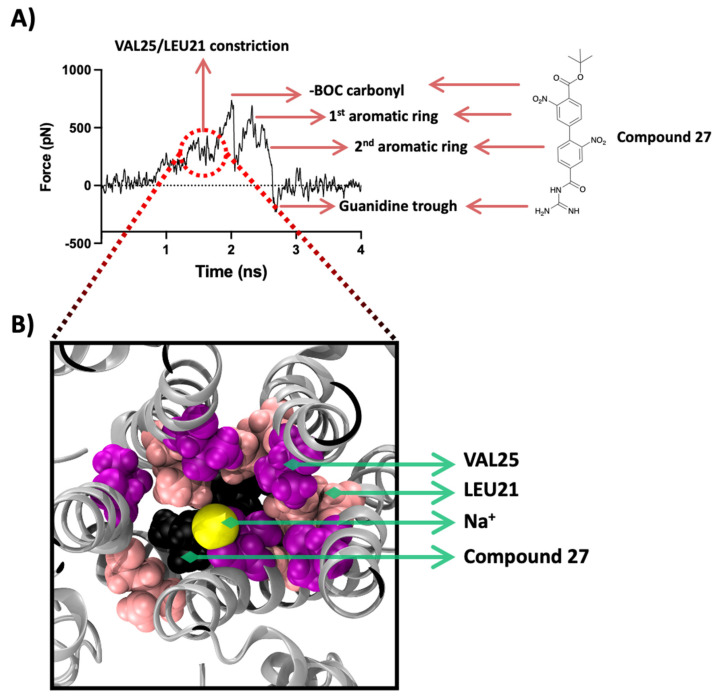
SMD results for compound 27. (**A**) SMD force profile (repeat #1) depicting the different peaks labelled by the respective molecular entity on the path of the SMD sodium ion. The first group of peaks represents the constriction formed by Val25/Leu21 followed by the highest peak formed by the interaction of the SMD ion with the BOC moiety of 27. The 3rd and the 4th peaks represent the obstacle formed by the two aromatic rings. The isoguanidine moiety of 27 leads to a trough formation. (**B**) Top-down view along the *Z*-axis of the protein representing the frame ~1.5 ns showing the interaction of the SMD ion with the 27’s BOC moiety (shown in black) and the constriction formed by Val25 (magenta colour) and Leu21 (shown in light pink), all shown in VDM representation with protein shown in silver cartoon representation. The sodium ion is shown in yellow as VDW representation.

The force profile plot of the Na^+^ SMD ion passing through the 27-bound E protein shows an initial barrier in the force profile (Figure 7A) that imposes resistance to the movement of the Na^+^ due to the presence of the gatekeeper-like residues, Leu28 and Val29, at about 0.8 ns into the simulation. A second hurdle is observed along the plane of Val25 and Leu21 with a magnitude of ~350–400 pN at about 1.6 ns. The following three energy peaks represent the interactions of the SMD Na^+^ with three distinct components of 27, i.e., BOC moiety and the two nitrobenzene aromatic rings. The first peak which is also the highest, at ~700 pN, is a result of obstruction formed by the BOC moiety. A snapshot of this interaction from the SMD simulation is shown in Figure 7B. The negatively charged carbonyl functional group interacts electrostatically with the positively charged Na^+^.

During the visual inspection of this SMD simulation, we observed that the -BOC carbonyl oxygen electrostatically interacts and coordinates its orientation with the movement of the Na^+^. This leads to an initial rotation of the BOC moiety, which is then immediately reversed after the Na^+^ passes through. This visual observation agrees with the SMD force profile where the interaction of the SMD ion and the BOC moiety results in the highest intensity peak followed by a trough when the Na^+^ is released from this electrostatic environment. The second and third peaks belong to the cation-π interactions of the Na^+^ with the two nitrobenzenes, as the Na^+^ is observed to coordinate its orientation with the plane of the aromatic rings in the structure of 27.

Similarly, the acylguanidine, which is a positively charged group, repels the positively charged SMD ion and leads to a trough in the plot or a push along the axis of movement, which creates a negative energy value at around 2.7 ns. Another interesting observation from this SMD study is that the barrier formed by the pore-lining residues in the presence of a strongly bound and bulky ligand such as 27 imposes a relatively lower energy barrier.

The peak formed by Val25 and Val21 sits at ~400 pN, whereas the same group of residues in the *apo*-state SMD simulation yielded a much higher intensity force constraint at ~600 pN (Figure 6A). This energy barrier reduction may result from changes in the conformation of the pore residues, imposed by allosteric effects of the ligand and may disrupt the functional properties of the pore such as ion selectivity and ion conduction. 

**Figure 8:**
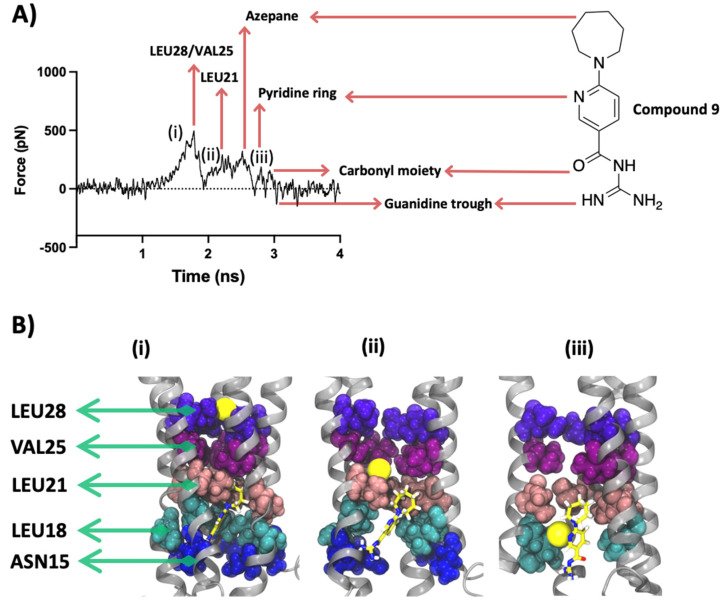
SMD results for compound 9. (**A**) SMD force profile (repeat #3) depicting the different peaks labelled by the respective molecular entity on the path of the SMD sodium ion. The first group of peaks represents the constriction formed by Leu28/Val25 followed by a smaller peak formed by the interaction of the SMD ion with Leu21. The remaining minor peaks are formed by the resistance imposed by different chemical moieties of 9, namely the azepane and pyridine rings and the isoguanidine group. (**B**) Lateral view along the *Z*-axis of the protein representing the significant ion interactions marked (i–iii) on the force profile. Protein is shown in silver cartoon representation and the residues are shown with VDW representation. The sodium ion is shown in yellow as VDW representation. Some chains and residues are excluded from the visualization for a better view.

Compound 9 is representative of the relatively weaker blockers in our data set, which also interacted with the same pore-lining residues as 27 (Figure 8).

The structural differences between 27 and 9 lie in the central ring, where the former has a more planar nitro-substituted aromatic ring and the latter bears a seven-membered unsubstituted heterocyclic ring. This heterocyclic ring is more flexible in nature and occupies a larger pocket-like space. As such, unlike 27, whose hydrophobic BOC moiety interacts non-electrostatically with the hydrophobic pocket formed by residues Leu21, Ala22, Val24, Val25 and Phe26, 9 fails to anchor its lipophilic end within the hydrophobic luminal residues and is thereby not sterically involved with the pore-lining residues. 

When the Na^+^ was pulled through the lumen of the compound 9-bound E protein, its movement was constrained initially by collective forces imposed by Val25 and Leu28 forming a similar gatekeeping effect. This force peaked at ~500 pN and was released to form a significant trough at 2 ns when the Na^+^ ion jumped into the central cavity (more about this is discussed in the pore analysis section). Leu21 also forms a low-intensity peak, briefly blocking the SMD ion passage, which is followed by the encounter of the Na^+^ ion with the ligand’s azepane and pyridine rings. The ion forms a cation-π interaction with the pyridine ring. Each of these interactions is followed by a trough and abolition of the exerted forces. This result indicates that the different individual chemical moieties of 9 are able to interact with the Na^+^, but the molecule as a whole is not capable of opposing the SMD forces which can be attributed to its higher free binding energy and weaker interactions with the protein. This is unlike 27, which has both a lower free binding energy and forms strong chemical interactions with the protein and does not allow the Na^+^ to be completely released (smaller troughs in the force profile, Figure 7) as a result of sequential interactions along the ion conduction pathway.

The SMD force profiles from the control group of amantadine, HMA and EIPA show that the ligand-related peaks are either absent or lower in intensity. This finding supports our free energy binding experiment in which the control group molecules were shown to be weakly bound to the protein lumen and most of the key interactions were absent. Figure S3 illustrates all the SMD force profiles of the eight systems and their three repeats.

### 2.6. Lumen Patency in the apo-State and in the Presence of Ligands

**Figure 9:**
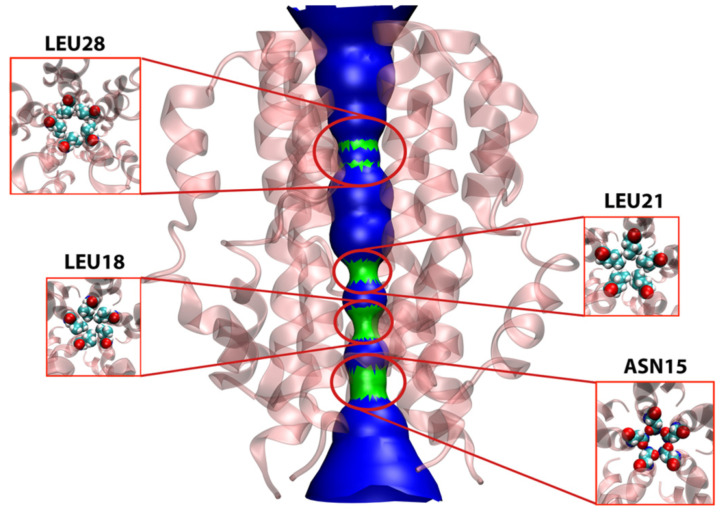
Pore analysis of E protein model in *apo*-state. The figure shows the surface presentation of the pore with emphasis on four main constrictions formed by pore-lining residues namely Leu28, Leu21, Leu18 and Asn15. The arrangements of these residues inside the pore are further magnified to show their pentameric arrangement responsible for the constriction they produce.

The HOLE2 program was used to analyze the pore dimensions of the E protein in both the unbound *apo*-state as well as bound to different groups of ligands, in this case, the same simulation systems that were studied in the SMD section. The unbound protein (Figure 9) shows four main points of constriction. Starting from the N-terminal end, Asn15, Leu18, Leu21, and Leu28 are responsible for limiting the pore diameter. These residues are responsible for selective permeability as well as dehydrating the ions that pass through the lumen, thereby playing key roles in the channel’s functional properties [19,20].

In the *apo*-state, the lumen of the pore acquires the widest radius close to the midline spanning residues 22 to 27 at 5 Å. This increase in diameter results in the formation of a so-called central cavity, a phenomenon quite commonly observed in other ion channels such as voltage-gated cationic channels [45,46]. This type of cavity is usually water filled and relatively hydrophobic in nature, allowing a lower electrostatic barrier to the passage of ions, and in general, an energetically favourable environment for ion conduction [47].

**Figure 10:**
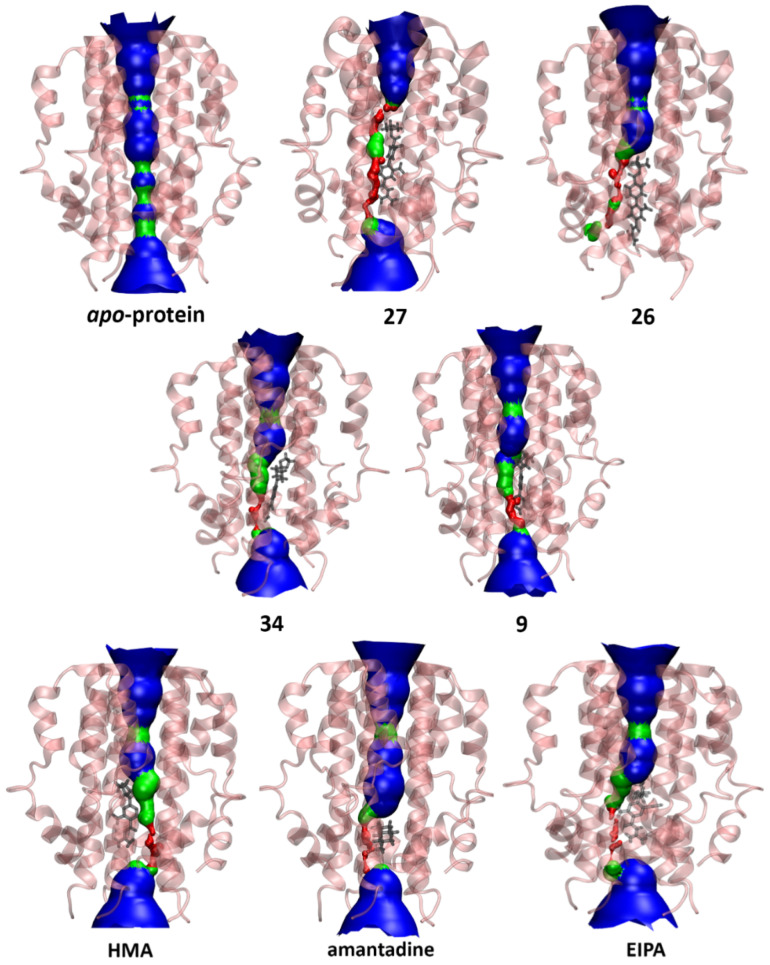
Pore analysis of E protein model in the *apo*-state and drug-bound state. The colour coding of the pore dimensions is based on the size of a water molecule, where red represents the pore radius being too constricted for a water molecule, green is where there is room for a single water molecule and blue colour is where the radius is double the minimum for a single water molecule to pass through.

Interestingly, this water-filled central cavity is conserved when the protein is bound to the majority of the ligands in our study with the exception of 27 (see Figure 10), which may be attributed to the elongated planar nature of this molecule positioning itself in the pore, spanning the distance between Glu8 and Val25 and exhibiting significant interactions with these residues (Figure 5). Additionally, upon visualization of MD trajectories, it was found that the distortion caused by the binding of 27 led to the abolition of water transfer throughout the 200 ns simulation. The plotted data from HOLE2 analyses are shown in Figure S4. 

## 3. Materials and Methods

### 3.1. Building an Updated Model of the SARS-CoV-2 E Protein

**Figure 11:**
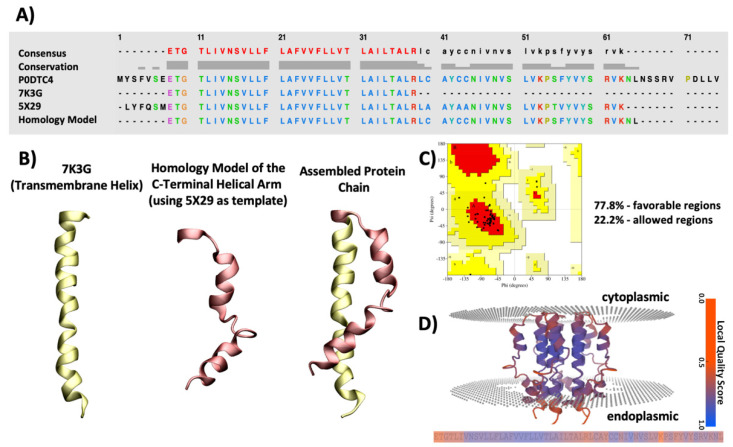
An overview of the SARS-CoV-2 protein E protein model development and assessment. (**A**) Sequence alignment of SARS-CoV-2 protein E showing the sequence of the entire E protein derived from UniProt database (PROT ID P0DTC4), sequence of 7K3G (NMR structure of the SARS-CoV-2 E protein), 5X29 (NMR structure of the SARS-CoV-1 E protein) and the final model length. (**B**) Cartoon presentation of the transmembrane helix derived from the PDB structure of SARS-CoV-2 E protein (7K3G), the homology model of the C-terminal helical arm using the PDB structure of SARS-CoV-1 (5X29) as the template and the assembled final model after fusing the two together. (**C**) Ramachandran plot of the final model showing 77.8% residues in the favourable region (red) and 22.2% of residues in the allowed region (yellow). (**D**) Final model with the predicted position within the membrane, coloured by Q-mean value using the QMEANBrane server. The QMean values range from 0 to 1.0 showing an array from red (lowest quality) to dark blue (highest quality). The model illustrates an acceptable range of QMean values.

The nucleotide sequence of the SARS-CoV-2 E protein was obtained from the NCBI GenBank (Accession code: NC_045512). For choosing the best three-dimensional structural template to build the homology model of the C-terminal helical arm, sequence alignments were performed in BlastP suite using the BLOSUM62 algorithm and an E value threshold of 10 × 10^−3^ [48]. Several homology models were built using I-TASSER [49], Modeller Suite [50], and SWISS-MODEL [51]. The Qualitative Model Energy Analysis (QMEANBrane) server [52] was used to aid in choosing the highest quality model. QMEANBrane is a tool comprising of statistical potentials targeted at the local quality estimation of membrane protein models after identifying the transmembrane region using an implicit solvation model. Additionally, Ramachandran plots were assessed which ultimately resulted in choosing the model from I-TASSER [53]. Figure 11A–D shows an overview of the SARS-CoV-2 E protein model development and assessment, further discussed in the following sections.

### 3.2. Small Molecule Docking

Our small molecule docking set is comprised of 49 input structures listed in Table S1. Compounds 9, 11, 22, 26, 27 and 33–38 are derived from our earlier study, where we designed and synthesized a library of HMA derivatives as potential inhibitors of influenza A/M2 ion channel [42]. To expand our ligand library, we also included HMA derivatives (compounds 39–62) previously reported as inhibitors of the human urokinase plasminogen activators [54]. Finally, the ligand library studied here also encompasses a set of other previously reported viroporin inhibitors (63–69, amantadine, rimantadine, HMA, EIPA, BIT225, BIT314 and M2WJ332) from the literature [55,56,57,58,59,60,61].

**Figure 12:**
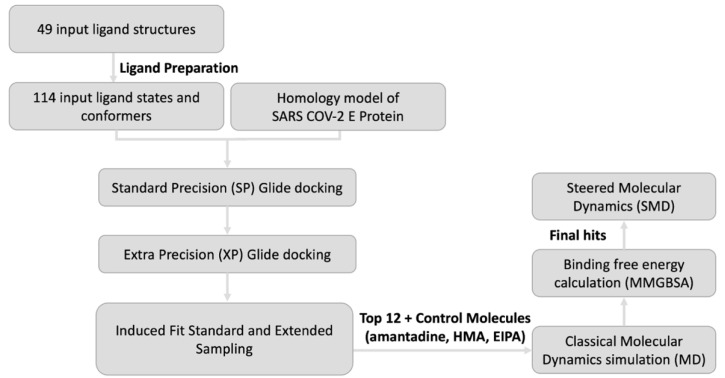
Modelling workflow. The workflow comprises of SP Glide docking, and XP Glide docking followed by pose refinement through IF Standard Sampling and IF Extended Sampling. The top 12 hits along with amantadine, HMA and EIPA were exposed to MD simulations followed by MM/GBSA free energy calculation. SMD simulation was performed for final hits to study the pore and ligand binding.

These starting structures were prepared using the LigPrep module of Schrodinger [62] using the OPLS3e forcefield, which yielded a total of 114 ligand states having different ionization and tautomeric states, torsional bond rotation angles and conformers. The ligands were desalted and the ionization states were assigned at a pH of 6.5 ± 1.0 using Epik [63,64]. Stereoisomers for each ligand were generated to a maximum of 3 states per input structure. Furthermore, the protein structure was prepared using the protein preparation wizard of Schrodinger. The docking protocol comprised of three main stages (Figure 12), namely Standard Precision (SP), Extra Precision (XP) and Induced Fit (IF) docking with standard and extended sampling. The docking search spaces for SP and XP docking calculations were chosen to include the intralumenal pore of the protein extending from Asn15 to Arg38 and were set at 20 Å^3^. All docking simulations were carried out using no ligand torsional constraints and using the flexible sampling mode. The van der Waals (VDW) radii scaling factor was set to 0.5 with a partial charge cut-off of 0.25. 

### 3.3. Classical MD Simulations

**Figure 13:**
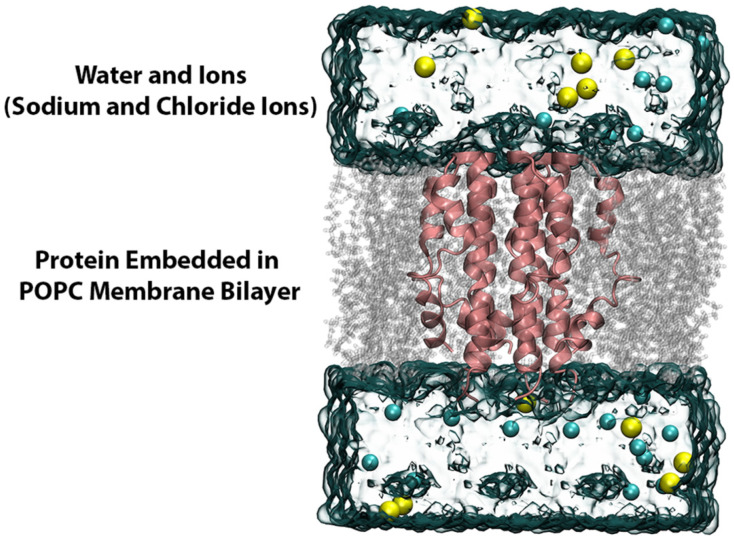
MD simulation setup. The MD simulation system was built by embedding the E protein model in a POPC lipid bilayer, further hydrated with water molecules and ions (Na^+^ and Cl^−^).

The resulting protein–drug complexes from the small molecule docking and the *apo*-protein system were further embedded in a lipid bilayer composed of POPC using the Membrane Builder program of CHARMM GUI [65,66,67,68,69]. Next, the tetragonal membrane system was hydrated with TIP3P water molecules on both lower and upper sides of the membrane along with sufficient Na^+^ and Cl^−^ ions to attain an overall ionic concentration of 150 mM. A visual representation of the entire system is shown in Figure 13. The parameters and topologies for the protein, lipids and ions were appointed using the CHARMM36m forcefield and the ligand topology and parameter files were generated using the ParamChem CHARMM General Force Field program, version 3.0.1 [70,71,72,73,74].

The MD simulations were performed using the NAMD package (version 2.14) [75] on Compute Canada’s Cedar, Graham and Beluga supercomputers. Initially, the standard CHARMM-GUI protocol of six minimization and equilibration stages totalling about 0.7 ns were performed. A force-based switching function was used for the van der Waals interactions to smoothly switch off between 10 and 12 Å. The bond lengths comprising hydrogen atoms were constrained using the SHAKE algorithm and Particle Mesh Ewald was used for computing the electrostatic interactions. A temperature of 303.15 K was assigned during the equilibration and production. NPT equilibrations were carried out for 250 ps by keeping the number of particles, pressure and temperature constant and the production MD simulations of the systems were carried out for 200 ns with a time step of 2 fs under periodic boundary conditions. The calculations of interactions are as follows: bonded interactions computed every 2 fs; short-range non-bonded interactions every 4 fs; and long-range electrostatic interactions every 8 fs. The Nosé-Hoover Langevin thermostat was employed to maintain constant temperature and pressure (1 bar) during the production simulations. 

### 3.4. Free Binding Energy Calculations

A total of 16 MD simulations, 200 ns in length, were performed in this study. Molecular Mechanics/Generalized Born Surface Area (MM/GBSA) module of AmberTools version 20.09 [43,44] was used to calculate the free binding energy of ligands in an ensemble of snapshots obtained from the 200 ns long MD simulations. Despite the higher level of accuracy often achieved by measuring free energy of binding based on the fundamental framework of computationally expensive explicit solvation models, for the purpose of evaluating the relative differences in free energy of binding amongst the different ligands under study, the solvation energies were calculated using the more computationally efficient Generalized Born implicit solvent model in AMBER and igb = 1 model, using snapshots of the system extracted from the MD production trajectory at every 300 ps. The final energy score reported is an average of the binding free energies of all the included snapshots.

### 3.5. SMD Simulations

**Figure 14:**
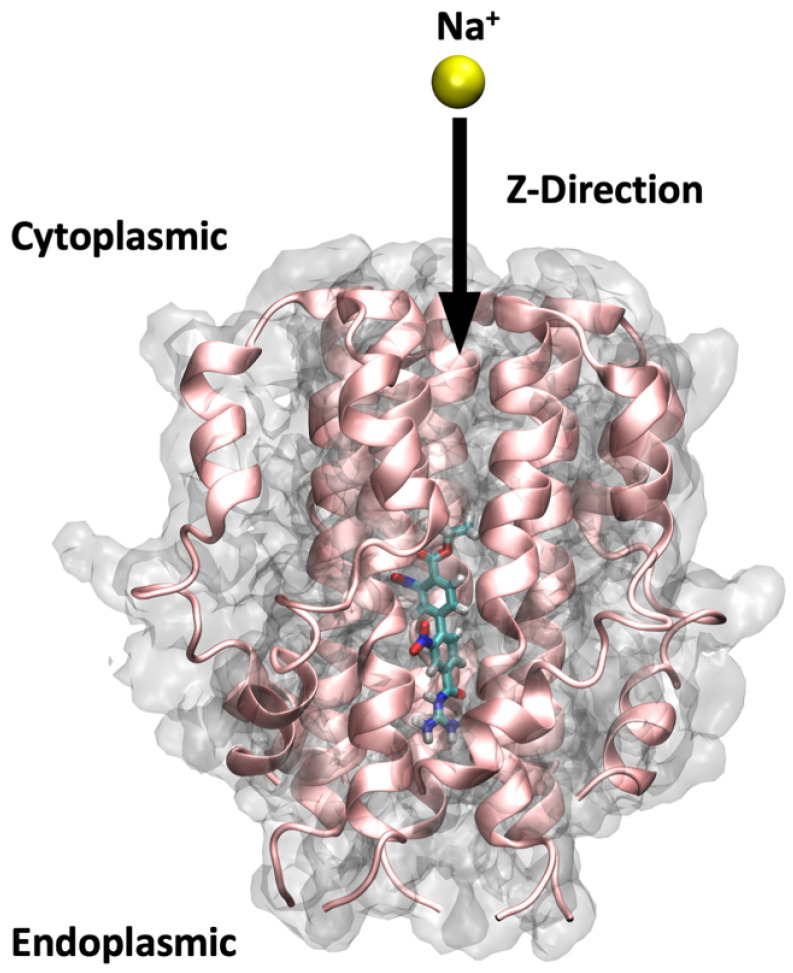
Steered molecular dynamics setup. The figure shows a ligand bound to the protein pore and illustrates the direction through which the sodium ion has been pulled in the SMD simulations of this study, i.e., from the C-terminal end to the N-terminal.

The SMD starting structures for the *apo*-protein and the protein–ligand complexes were obtained from the equilibrated section of the MD trajectories as per the Root Mean Square Deviation (RMSD) analysis. Similar constraints to those used during MD were maintained during the SMD simulations using the NAMD 2.14 package. As depicted in Figure 14, our SMD simulations were carried out along the *Z*-axis by pulling a Na^+^ from the C-terminal end of the TM helix to the periphery of the N-terminal for a total duration of 4 ns. To ensure the stability of the protein and membrane systems during the ion pulling process, four C-alpha atoms in the backbone of the protein (Ile33) located in the transmembrane helix remained constrained with a force of 1 kcal/mol. A spring constant of 4 kcal/mol/Å and a constant velocity of 0.015 Å/ps were employed for the pulling of the Na^+^. The SMD simulation parameters were calibrated following several test runs to ensure that the passage of the SMD ion neither destabilized the pentameric structure arrangement of the protein nor caused significant displacement of the ligand from its binding site. Three repeats for each of the ligand-bound protein systems, and 6 repeats for the *apo*-protein system were performed to ensure reproducibility of the results, totalling 24 SMD trajectories.

### 3.6. Analysis and Visualization

RMSD and Root Mean Square Fluctuation (RMSF) analyses were carried out using the CPPTRAJ module [76] in AmberTools version 20.09. Visualizations and trajectory analysis were performed in Visual Molecular Dynamics (VMD) [77] and Chimera suite [78]. Pore radius profiles were calculated using the HOLE2 program [79]. All the plots included herein were generated using GraphPad Prism version 9.0. (GraphPad Software, La Jolla CA, USA, www.graphpad.com).

## 4. Conclusions

The identification of a gap in the literature regarding the unavailability of a complete protein structure for the SARS-CoV-2 E protein motivated us to construct a comprehensive model that incorporated both the functionally important channel components, i.e., the TMD as well as the C-terminal helical arm. This model provided us with a platform to delve into the atomic level details of both channel properties and potential ligand interactions.

Utilizing small molecular docking and classical MD simulations, we studied the binding and dynamic behaviour of a panel of compounds comprised of HMA, its derivatives and other control molecules. This enabled us to derive several key findings related to the atomic level interactions of the ligands inside the lumen of the E protein and our findings support the significance of pore-lining residues such as Glu8, Thr11, Asn15, Leu18, Leu21, Val25 and Leu28 for ligand binding.

SMD simulations were performed on the E protein in the unbound and bound states for selected ligands divided into three categories based on binding free energy results: strongly bound ligands (26 and 27), weakly bound ligands (9 and 34) and control group (amantadine, HMA and EIPA). Na^+^ was pulled through the intralumenal space from the C-terminal of the TM helix to the N-terminal end of the channel during the simulation and aided in revealing the various high-energy barriers that could be responsible for ionic conduction but also small molecule interactions. The data from the SMD analyses offer valuable insights as to what type of chemical moieties and chemophoric properties can: (i) potentially alter the characteristics of the pore; (ii) form energetic barriers against ionic conduction; and therefore, (iii) act as channel blockers/inhibitors. Similarly, we were able to confirm that 27 from our dataset binds to the E protein with an acceptable free binding energy and forms interactions with a wide range of residues throughout the MD simulations.

This ligand, when bound to the E protein, acts as a robust blockade against the path of the pulled Na^+^ during the SMD simulations as compared to the weaker blockers or the control molecules in our study. Moreover, the binding of 27 altered the typically observed energy barriers of the residues such as Leu28 that were otherwise present at higher values in the *apo*-state, pointing to a semi-allosteric effect. Based on these experimental results, we inferred that an ideal inhibitor of the E protein should be elongated, linear and planar in its chemical scaffold, bear a hydrophobic end capable of probing non-electrostatic interactions as well as a hydrophilic end capable of forming hydrogen bonds.

Collectively, the information presented here primarily validates the functionality of our SARS-CoV-2 E protein comprehensive model as a computational tool to aid future in silico studies on this crucial viral target and highlights the importance of HMA derivatives as potential inhibitors of SARS-CoV-2 E protein to guide the development of subsequent small molecule inhibitors.

## Figures and Tables

**Table 1:** List of the top 12 molecules along with control molecules (amantadine, HMA and EIPA). The table lists the structure of the molecules along with their IFD Glide docking scores which were sorted by free binding energies from MM/GBSA calculations in ascending order.

Compound Number/Name	Structure	IFD Score (Kcal/Mol)	MM/GBSA Free Binding Energy (Kcal/Mol)	Cell Survival MTT Assay Estimated CC_50_ (µM)
26	** 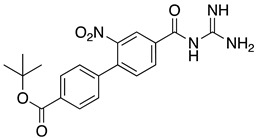 **	−10.02	−40.75	25 ± 5 [42]
27	** 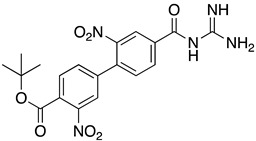 **	−9.35	−38.96	55 ± 17 [42]
61	** 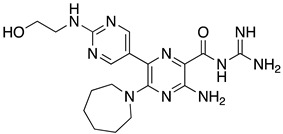 **	−7.62	−35.65	
33	** 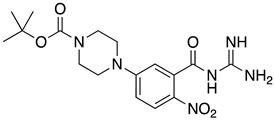 **	−8.16	−32.82	
37	** 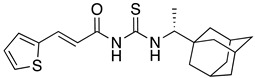 **	−6.94	−28.99	
EIPA	** 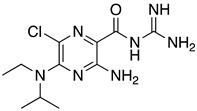 **	−4.03	−27.51	
49	** 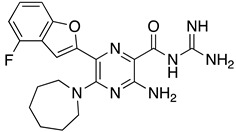 **	−6.36	−26.82	
HMA	** 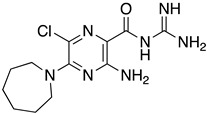 **	−5.32	−25.46	4.7 ± 0.3 [42]
52	** 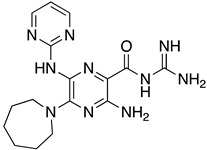 **	−5.54	−25.07	
43	** 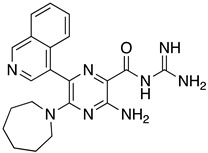 **	−4.86	−24.39	
48	** 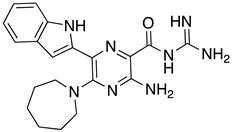 **	−6.77	−22.11	
62	** 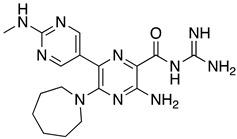 **	−5.25	−20.03	
34	** 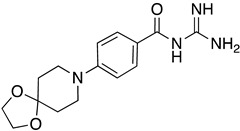 **	−4.93	−11.21	
9	** 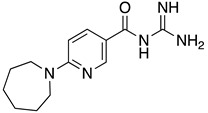 **	−4.54	−10.98	>100 [42]
amantadine	** 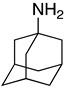 **	−3.66	−8.67	>100 [42]

## Data Availability

The datasets generated during this study are available upon request from the corresponding author.

## Supplementary Materials

The supporting information can be downloaded at: https://www.mdpi.com/article/10.3390/ijms231810647/s1.

## Abbreviations

BOC, tert-butyloxycarbonyl; EIPA, N-ethylisopropylamiloride; ERGIC, endoplasmic-reticulum-Golgi intermediate compartment; HMA, Hexamethylene Amiloride; IFD, Induced Fit docking; MD, Molecular Dynamics; MERS, Middle East Respiratory Syndrome; MM/GBSA, Molecular Mechanics/Generalized Born Surface Area; NMR, Nuclear Magnetic Resonance; RMSD, Root Mean Square Deviation; RMSF, Root Mean Square Fluctuation; SARS, Severe Acute Respiratory Syndrome; SMD, Steered Molecular Dynamics; ssNMR, Solid State Nucleic Magnetic Resonance; TMD, Transmembrane Domain.
