# Supplementary material for: In Silico Evaluation of Hexamethylene Amiloride Derivatives as Potential Luminal Inhibitors of SARS-CoV-2 E Protein
Source: Int J Mol Sci. 2022 Sep 13;23(18):10647. doi: 10.3390/ijms231810647 (PMC9503309; doi:10.3390/ijms231810647)
Supplement: Supplementary file 1 [file ijms-23-10647-s001.zip › ijms-1856725-supplementary.pdf]

Supplementary Data

Table S1. The panel of small molecules screened against SARS-CoV-2 E protein.

Compound Name/Number	Structure	XP Docking Score	Glide Induced Fit Docking
**amantadine**	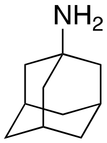	-2.257	-3.995
**rimantadine**	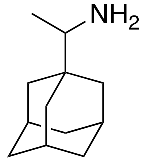	-3.885	-5.046
**HMA**	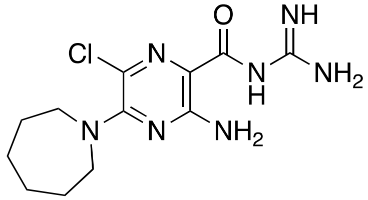	-6.246	-5.326
**EIPA**	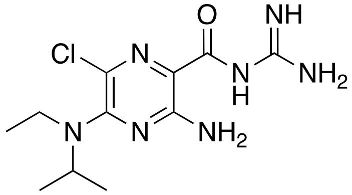	-7.878	-4.905
**BIT225** [55]	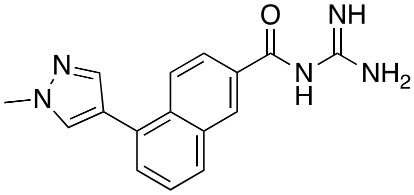	-4.560	-7.515
**BIT314** [55]	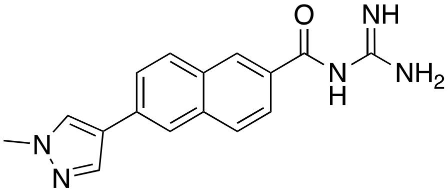	-5.445	-7.796
**63**	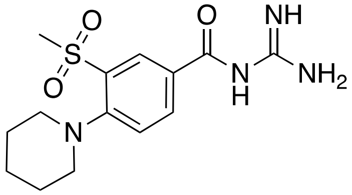	-5.664	-5.989

Compound Name/Number	Structure	XP Docking Score	Glide Induced Fit Docking
**64** [56]	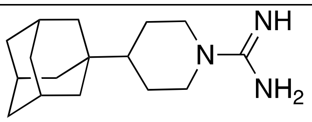	-7.720	-7.399
**M2WJ332** [56]	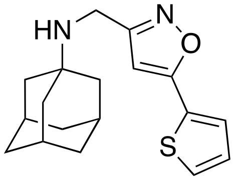	-5.841	-7.100
**69** [56]	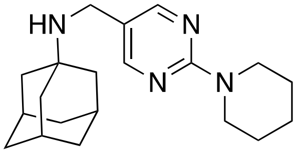	-4.543	-6.742
**65** [60]	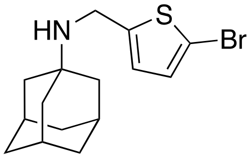	-4.734	-5.842
**66** [59]	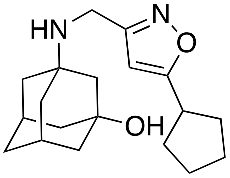	-5.713	-8.355
**67** [57,58]	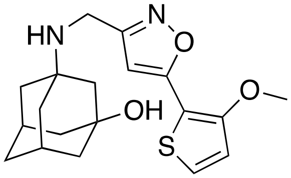	-5.709	-7.264
**68**	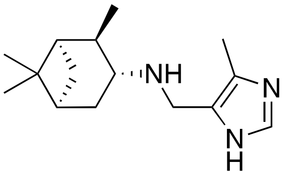	-4.251	-5.025
**9**	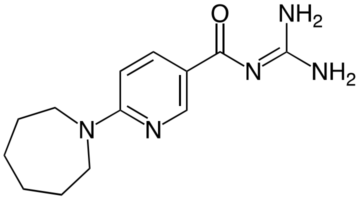	-5.541	-6.420

Compound Name/Number	Structure	XP Docking Score	Glide Induced Fit Docking
**11**	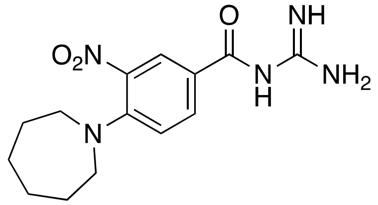	-5.588	N/A
**34**	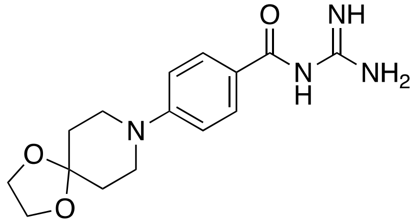	-3.711	-6.311
**35**	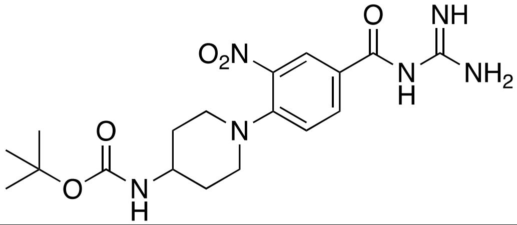	-4.201	-6.592
**36**	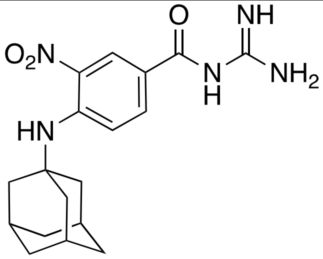	-4.894	-5.322
**37**	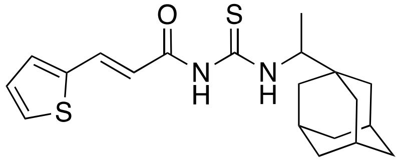	-4.769	-8.507
**33**	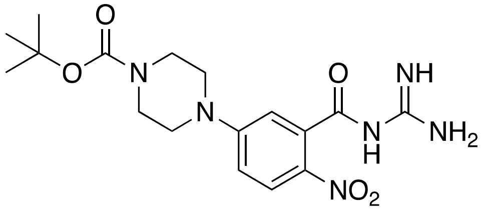	-8.201	-5.576

Compound Name/Number	Structure	XP Docking Score	Glide Induced Fit Docking
**22**	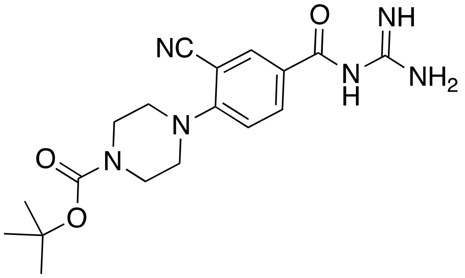	-5.449	-6.643
**38**	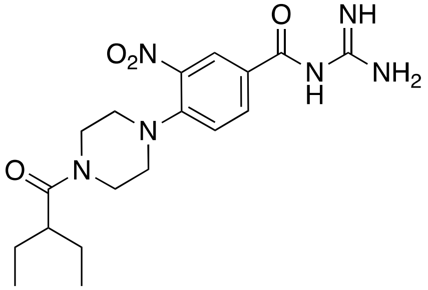	-3.288	-6.280
**26**	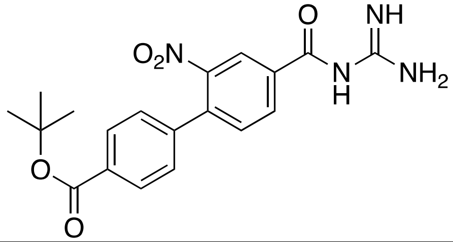	-5.285	-7.397
**27**	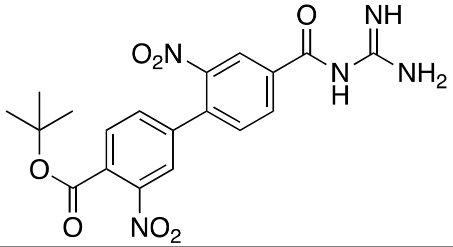	-4.533	-6.353
**39**	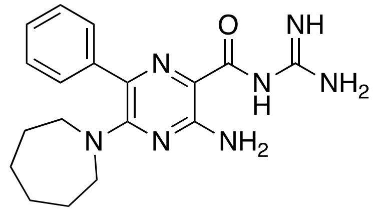	-4.556	-6.591
**40**	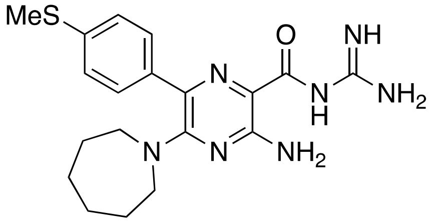	-4.271	-6.871

Compound Name/Number	Structure	XP Docking Score	Glide Induced Fit Docking
**41**	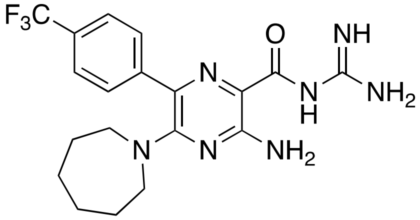	-2.736	-5.638
**42**	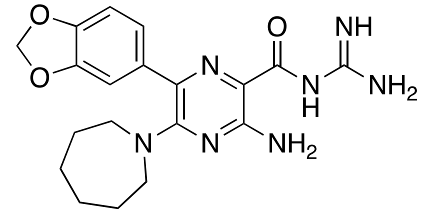	-4.288	-6.307
**43**	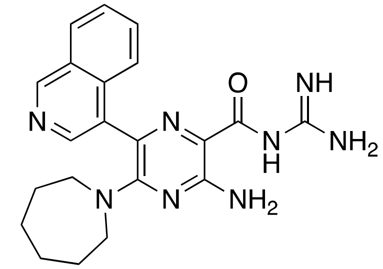	-4.808	-6.624
**44**	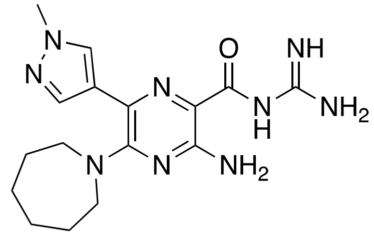	-3.844	-4.271
**45**	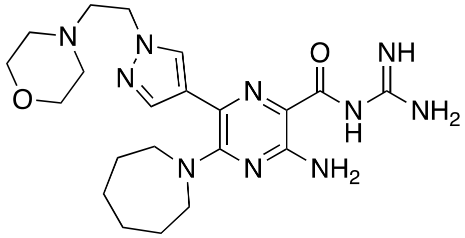	-3.005	-4.810
**46**	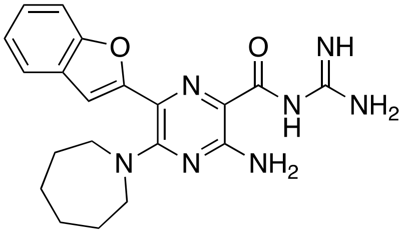	-2.578	-5.480

Compound Name/Number	Structure	XP Docking Score	Glide Induced Fit Docking
**47**	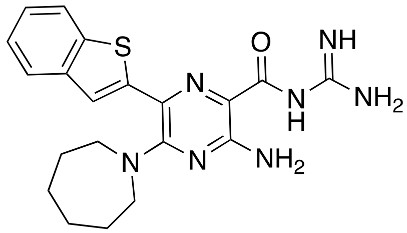	-2.936	-5.290
**48**	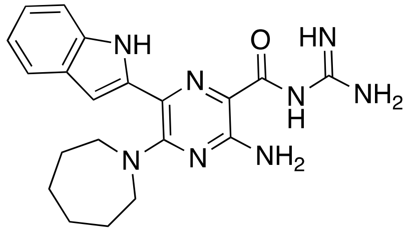	-4.403	-5.305
**49**	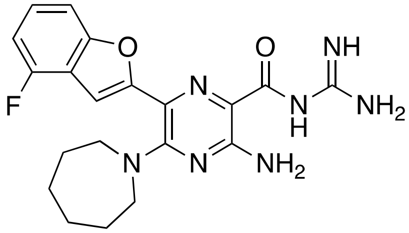	-5.020	-6.367
**50**	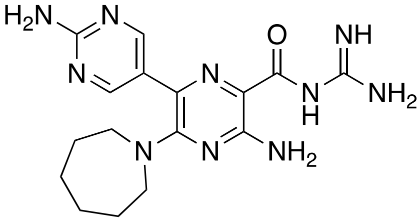	-4.105	-7.8
**51**	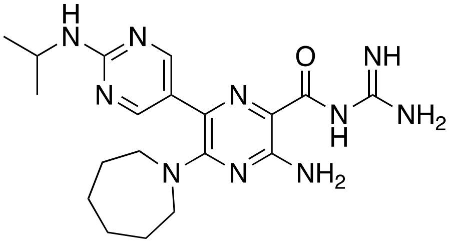	-3.214	-3.886
**52**	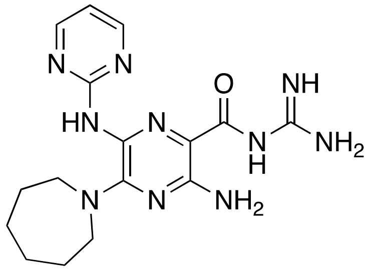	-4.531	-6.591

Compound Name/Number	Structure	XP Docking Score	Glide Induced Fit Docking
**53**	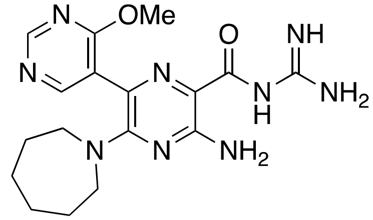	-4.112	-5.539
**54**	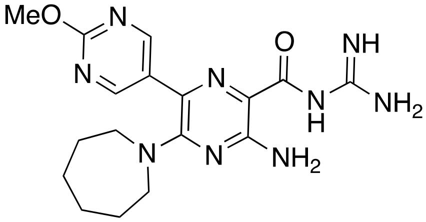	-4.112	-7.129
**55**	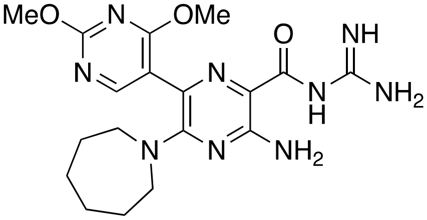	-3.866	-3.198
**56**	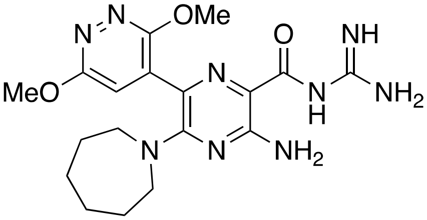	-2.696	-5.785
**57**	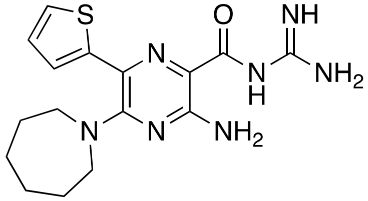	-3.689	-6.810
**58**	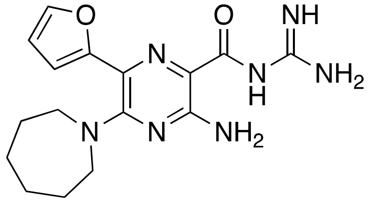	-4.144	-6.359
**59**	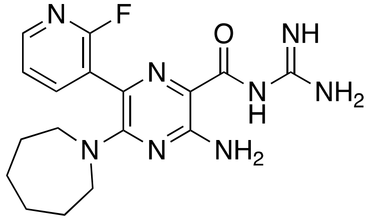	-4.106	-4.791

Compound Name/Number	Structure	XP Docking Score	Glide Induced Fit Docking
**60**	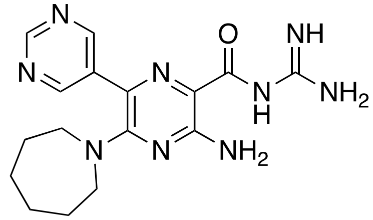	-4.556	-9.159
**61**	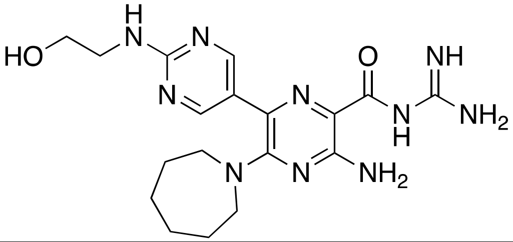	-5.375	-7.621
**62**	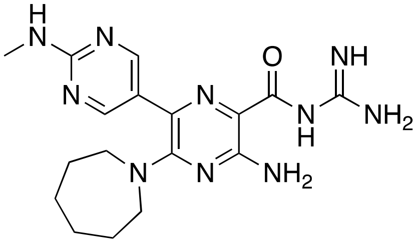	-4.548	-7.280

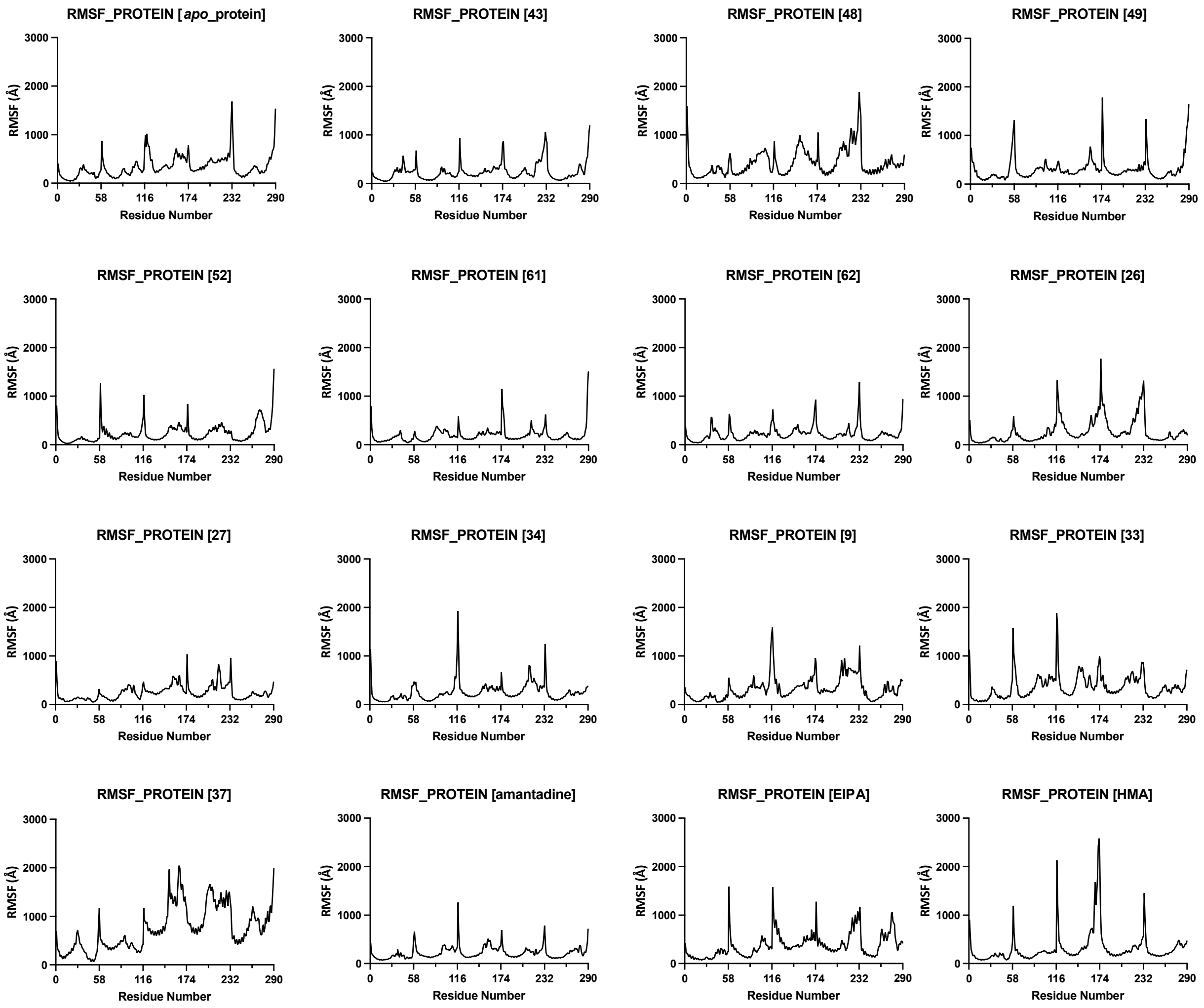

Figure S1. RMSF graphs of the five chains of the protein systems.

The presentation of residue numbers are sequential and the graphs are labelled by the respective bound-ligands. Highest degree of fluctuation observed at the C-terminal end of the TM helix of each monomer within the protein-ligand complex. The number in the graph title refers to the compound number from Table 1.

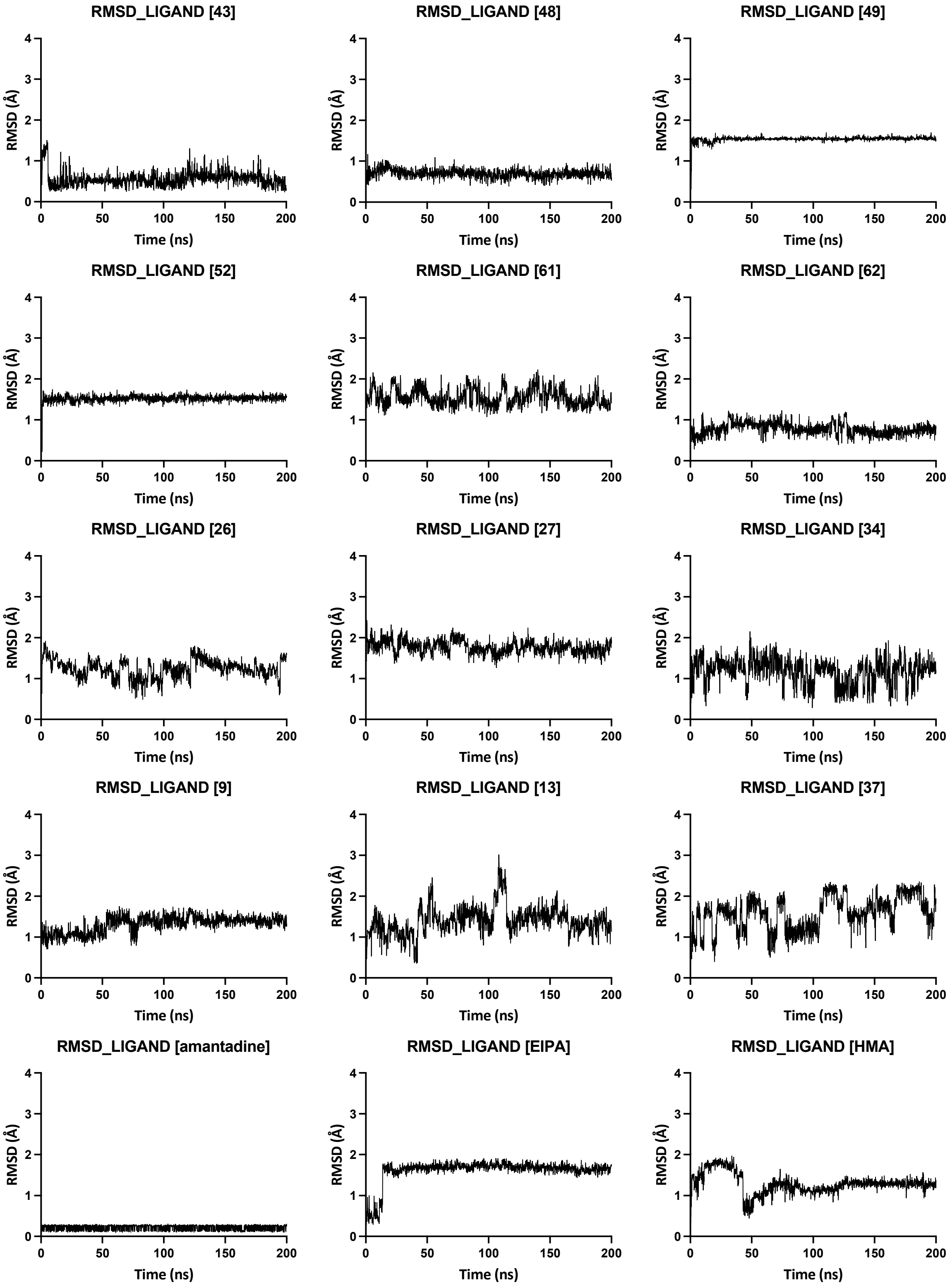

Figure S2. RMSD graphs of the ligands during MD simulations.

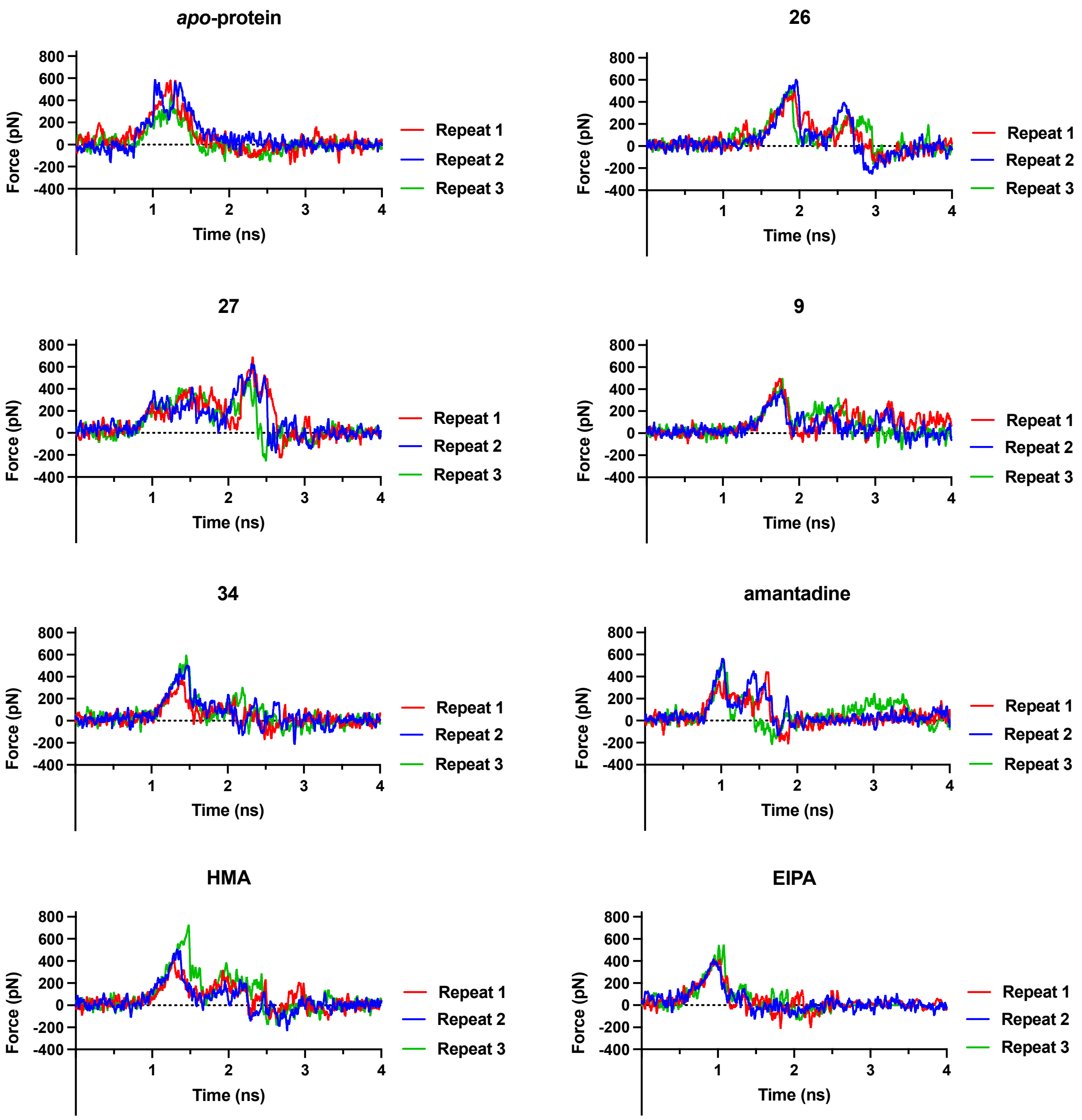

Figure S3. SMD force profile of a sodium ion pulled through the ligand-bound systems.

Three replicates are shown labelled by ligand-bound to each protein.

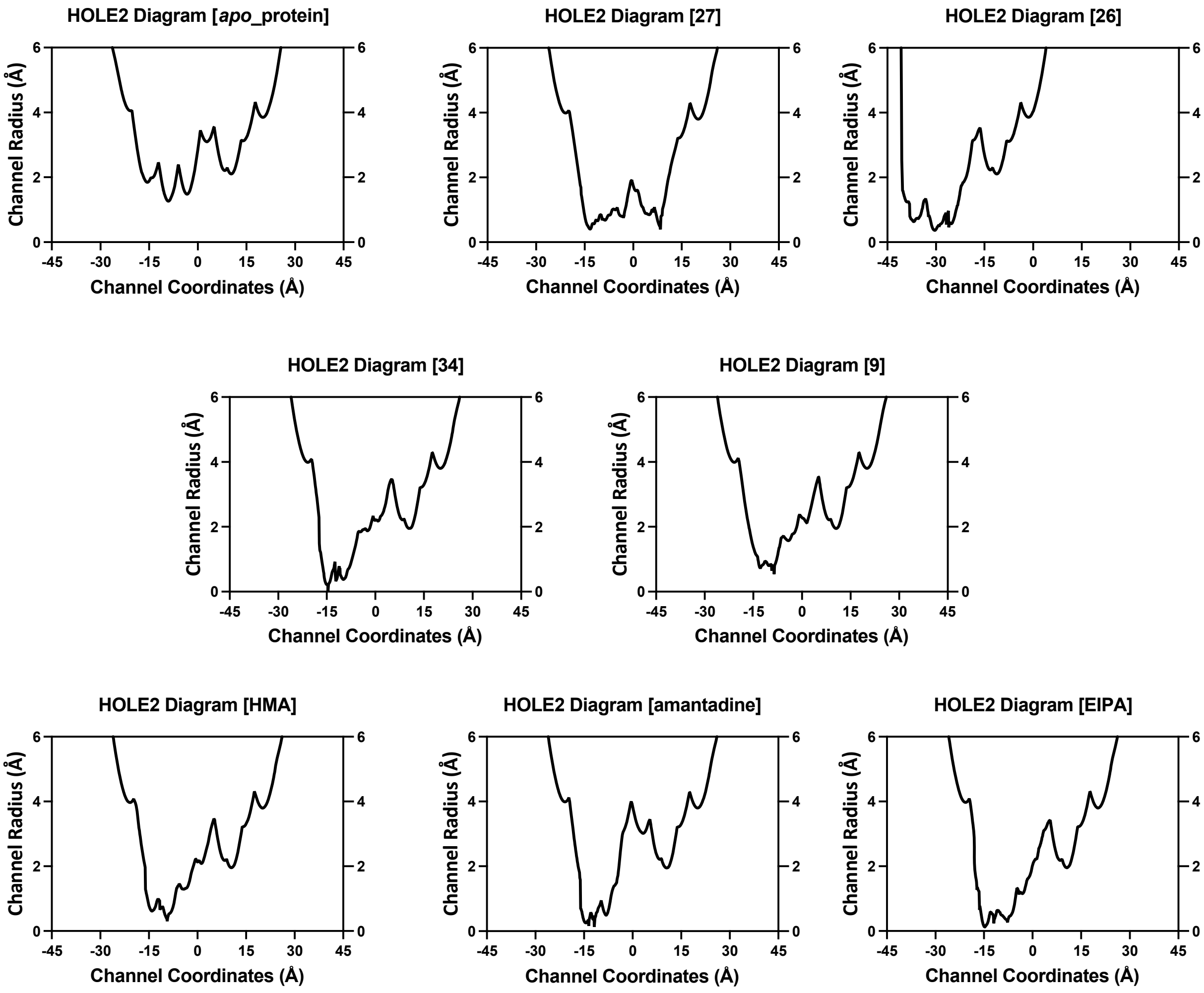

Figure S4. HOLE2 analysis diagram of protein systems bound to ligands.

X-axis represents channel coordinate (Å) and Y-axis shows the channel pore radius (Å) throughout the TMD along Z-axis of the protein. The graphs are titled by the compound number bound to the protein.
